# Optimization of cellulase production by *Penicillium* sp.

**DOI:** 10.1007/s13205-016-0483-x

**Published:** 2016-08-09

**Authors:** H. N. Prasanna, G. Ramanjaneyulu, B. Rajasekhar Reddy

**Affiliations:** Department of Microbiology, Sri Krishnadevaraya University, Anantapuramu, Andhra Pradesh India

**Keywords:** β-Glucosidase, Endoglucanase, Exoglucanase, Nutrients, Optimization, *Penicillium* sp.

## Abstract

The production of cellulolytic enzymes (β-exoglucanase, β-endoglucanase and β-glucosidase) by *Penicillium* sp. on three different media in liquid shake culture conditions was compared. The organism exhibited relatively highest activity of endoglucanase among three enzymes measured at 7-day interval during the course of its growth on Czapek-Dox medium supplemented with 0.5 % (w/v) cellulose. Cellulose at 0.5 %, lactose at 0.5 %, sawdust at 0.5 %, yeast extract at 0.2 % as a nitrogen source, pH 5.0 and 30 °C temperature were found to be optimal for growth and cellulase production by *Penicillium* sp. Yields of Fpase, CMCase and β-glucosidase, attained on optimized medium with *Penicillium* sp. were 8.7, 25 and 9.52 U/ml, respectively with increment of 9.2, 5.9 and 43.8-folds over titers of the respective enzyme on unoptimised medium. Cellulase of the fungal culture with the ratio of β-glucosidase to Fpase greater than one will hold potential for biotechnological applications.

## Introduction

Energy and environment are the essential aspects of human life almost all over the world. The conventional sources that meet the demand on energy needs will not last long and therefore non-conventional alternative and renewable sources are to be exploited for this purpose (Tuo 2013; Zhao et al. 2016). This planet is threatened due to environmental pollution in recent years as a result of disposal of solid and liquid wastes rich in organics. Solid and liquid waste rich in organics can be considered for generation of energy by biotechnological means (Koneswaran and Nierenberg 2008; Ashfaq and Khatoon 2013; Lytle 2016). Utilization of solid and liquid wastes will provide twin benefits saving the environment from polluted menace and generating energy (Hinds 2015; Jeihanipour and Bashiri 2015).

Cellulose constitutes bulk of the plant cell wall materials and is the most abundant and renewable non-fossil carbon source on earth (Li et al. 2009). Cellulose occurs in municipal wastes, forest products, agriculture, fruits and vegetables. Cellulose has enormous potential as a renewable source of energy (Coral et al. 2002) and a number of microorganisms use it as a carbon source. Major constraints in enzymatic hydrolysis of cellulosic materials for the production of fermentation sugar are low productivity and the high cost of cellulases (Lee et al. 2010). This cellulose polymer could be converted into simple sugars in saccharification process by cellulase enzymes derived from microbial system. Cellulase is a complex of three types of enzymatic complexes namely, cellobiohydrolases (EC 3.2.1.91), endoglucanases or CMCases (EC 3.2.1.4) and β-glucosidases (EC 3.2.1.21), acting synergistically to convert complex carbohydrates present in lingocellulosic (LC) biomass into glucose (Iqbal et al. 2011). The simple sugar can then be utilised by other organisms to produce a variety of fermentation chemicals (alcohols-ethanol, butanol, solvents-acetone or 2,3-butanediol etc.) (Gadgil et al. 1995; Hoshino et al. 1997; Van Wyk 2001; Lee et al. 2008; Sanil et al. 2015). In addition to their importance in saccharification, cellulases are currently being used in several other industrial processes—starch processing, animal feed applications, grain alcohol fermentation, malting and brewing, extraction of fruit and vegetable juices, paper and pulp industry, textile industry and waste water treatment (Bhat and Bhat 1997; Bhat 2000; Penttila et al. 2004; Koomnok 2005; Kuhad et al. 2011; Yano et al. 2012; Adrio and Demain 2014). In view of biotechnological importance, microbial production of cellulases continues to be a subject of interest and to attract a great deal of attention from cross sections of scientists. Secretion of cellulolytic enzymes by different organisms in nature needs to be continuously monitored. *Trichoderma* species, in particular, *reesei* received more attention for the production of cellulolytic enzymes but β-glucosidase activity was very low in cellulase enzymes of *Trichoderma* *reesei* (Peterson and Nevalainen 2012). Continuous search for the production of cellulase with high β-glucosidase activity has been bringing other organisms including *Penicillium* species into lime light (Gusakov and Sinitsyn 2012). The present investigation reported secretion of cellulolytic enzymes by a local and potential isolate *Penicillium* sp. grown on different nutrient sources in submerged fermentation in a laboratory study.

## Materials and methods

### Media

Composition of three media (basal medium, minimal medium and Czapek-Dox medium) used in this study is as follows and is expressed in g/L. Basal medium contained ingredients-yeast extract 10, NaCl 2, CaCl_2_ 0.2, KH_2_PO_4_ 2, FeCl_2_ 0.01, MgSO_4_ 1.7, NH_4_Cl 2, distilled water 1000. Minimal medium: KH_2_PO_4_ 0.04, K_2_HPO_4_ 0.1, Na_2_HPO_4_ 0.10, NH_4_SO_4_ 0.008, MgSO_4_ 0.02, (NH_4_)_2_SO_4_ 0.04, CaCl_2_ 0.027, distilled water 1000 and Czapek-Dox medium: sucrose 30, NaNO_3_ 2, K_2_HPO_4_ 1, MgSO_4_ 0.05, KCl 0.5, FeSO_4_ 0.01, distilled water 1000. pH of all the three media was adjusted to 7.0.

### Culture conditions and enzyme production

*Penicillium* sp., isolated from soil polluted with effluents discharged by a cotton ginning industry (Narasimha et al. 1999), was used in this study. Sterilised 50 ml of each of the three different media, (minimal, basal and Czapek-Dox) amended with 0.5 % cellulose as carbon source was distributed into sterile 250 m1 Erlenmeyer flask. Meanwhile, the spore suspension was prepared in sterile distilled water from 6-day-old culture of *Penicillium* sp. grown on Potato-Dextrose Agar (PDA) slants. The flasks were inoculated with a density of 2 × 10^6^ spores and incubated at 28 °C on a rotary shaker (140 rpm). As the maximal cellulolytic activity with fungal cultures was observed on the 7th day of incubation in the preliminary study (Prasanna 2003), flasks were withdrawn only on the 7 day of incubation and filtered through Whatman No. 1 filter paper to separate mycelial mat and culture filtrate.

Fungal growth was expressed in terms of dry weight (mg/flask) of mycelial mat after drying at 70 °C in an oven until constant weight. The content of soluble protein in the culture filtrate was estimated according to the method of Lowry et al. (1951) with bovine serum albumin as a standard. Total activity of cellulase complex and/or individual component enzyme activities in the culture filtrate were determined as per procedures described below. In view of maximum growth and cellulase activity on the Czapek-Dox medium at 7th day interval, subsequent experiments were carried out on Czapek-Dox medium, to find out the influence of supplementation of different carbon, nitrogen lignocellulose sources, temperature, pH and surfactants on growth, secretion of extracellular protein content and cellulase production by *Penicillium* sp. at only 7th day incubation.

### Enzyme assays

#### Fpase assay

Filter paper activity (FPA) for total cellulase activity in the cultural filtrate was determined according to the method of Mandels and Weber (1969). Aliquots of appropriately diluted culture filtrate as enzyme source was added to Whatman No. 1 filter paper strip (1 × 6 cm, 50 mg) immersed in 1 ml of 0.05 M sodium citrate buffer of pH 4.8. After incubation at 50 °C for 1 h, the reducing sugar released was estimated by Dinitrosalicylic acid (DNS) method (Miller 1959). One unit of filter paper (FPU) activity was defined as the amount of enzyme releasing 1 µmol of reducing sugar from filter paper per ml per minute.

#### CMCase assay

Endoglucanase activity carboxymethylcellulase (CMCase) was measured as described previously (Ghosh 1987) using a reaction mixture containing 1 ml of 1 % carboxymethyl cellulose (CMC) in 0.2 M acetate buffer (pH 5.0) and aliquots of suitably diluted filtrate. The reaction mixture was incubated at 50 °C for 30 minutes and the reducing sugar produced was determined by DNS method. One unit (IU) of endoglucanase activity was defined as the amount of enzyme releasing 1 µmol of reducing sugar per min.

#### β-Glucosidase assay

β-Glucosidase activity was assayed by the method of Herr (1979). β-glucosidase activity was measured in 1 ml of 5 mM *p*-nitropheny1-β-D-glucopyranoside (PNPG) in 0.2 M acetate buffer (pH 5.0) and aliquots of appropriately diluted culture filtrate and incubated at 50 °C for 30 min. The reaction was terminated by addition of 4 ml of 0.05 M NaOH–glycine buffer (pH 10.6) solution and the released *p*-nitrophenol was read at 405 nm and the activity was expressed in terms of liberation of *p*-nitrophenol from *p*-nitrophenyl-β-D-glucopyranoside (PNPG). One unit of the enzyme activity was defined as the amount of enzyme producing 1 µmol of *p*-nitrophenol per min.

## Results and discussion

*Penicillium* sp. was cultured on three liquid media (minimal, basal and Czapek-Dox) amended with 0.5 % cellulose at 28 °C under shaking conditions. Growth, extracellular protein content and total cellulolytic activity in the culture filtrate were monitored on 7th-day incubation and are presented in the Table 1. Czapek-Dox medium supported the maximum growth of *Penicillium* sp. when compared to basal and minimal media. The maximal secretion of extracellular protein content was derived from basal medium followed by Czapek-Dox medium and minimal medium; *Penicillium* sp. grown in Czapek-Dox medium yielded highest production of all three enzyme components of cellulase with 0.94, 4.21 and 0.21 U/ml filter paperase, Carboxymethyl cellulase and β-glucosidase, respectively. FPase activity was not detected in culture filtrate of basal medium but activities of CMCase and β-glucosidase to the titer of 2.88 and 0.19 U/m1 were observed. Minimal medium induced least activity of β-glucosidase, but no FPase and CMCase was detected. Cellulase activity was observed upon growth of *Penicilium* sp. on only mineral medium supplemented with avicel, rice straw, and CM-Cellulose but not on potato dextrose broth supplemented with the same cellulosic materials and reached maximum level with CMCase titers of 6–7 U/ml in 24 days incubation (Picart et al. 2007). Species of *Penicillium* (wild type) such as *pinophilum* (Jorgensen et al. 2005) *decumbens* (Sun et al. 2008) and *xanthinellum* (Singhvi et al. 2011) secreted about 1–3 U/ml of Fpase and β-glucosidase on cellulosic materials in submerged fermentation (SmF).

**Table 1:** Growth, protein secretion and cellulase production by *Penicillium* sp. on different media

Medium	Dry weight of mycelial mat (mg/flask)	Protein content (mg/ml)	Cellulase		
			^a^FPase (FPU/ml)	^b^CMCase (U/ml)	^c^Β-glucosidase (U/ml)
Basal	410	2.69	-nd-	2.88	0.19
Czapeck dox	450	1.70	0.94	4.21	0.21
Minimal	260	0.60	-nd-	-nd-	0.03

Values represented in the table are averages of results of two experiments

*-nd-* not detected

^a^Filter paperase (FPase) is expressed in terms of filter paper units. One unit is the amount of enzyme in the culture filtrate releasing µmol of reducing sugar from filter paper per min

^b^Carboxymethyl cellulase (CMCase) is expressed in terms of units. One unit is the amount of enzyme releasing 1 µmol of reducing sugar from carboxymethyl cellulose per min

^c^One unit of β-glucosidase activity is defined as the amount of enzyme liberating 1 µmol of *p*-nitrophenol per min

The major components of production medium like carbon and nitrogen sources and physical parameters like temperature, pH and incubation time were found to be critically affecting the cellulase production hence need to be optimized for every isolate (Polyanna et al. 2011). Influence of supplementation of different soluble carbon sources (0.5 %) to Czapek-Dox medium on cellulase production by *Penicillium* sp. was examined and is presented in Table 2. Among carbon sources, lactose was the best source followed by carboxymethyl cellulose and galactose for cellulase production, biomass and secretion of extracellular protein by *Penicillium* sp. Activities of even other individual components of cellulase, such as filter paperase and β-glucosidase were also highest in culture filtrate of *Penicillium* sp. grown in the presence of lactose. This study substantiates the work of Kathiresan and Manivannan (2006) and Devanathan et al. (2007) who demonstrated lactose as best inducer of *Aspergillus* sp. Muthuvelayudham and Viruthagiri (2006) reported maximum growth and cellulase enzyme production by *T. reesei* C5 with provision of lactose as sole carbon source. Similarly, lactose present in cheese and whey induced the cellulase biosynthesis in *Trichoderma reesei MCG 80* (Sternberg and Mandels 1979; Allen and Andreotti 1982). The presence of sorbitol at 0.5 % level along with cellulose supported maximum production of FPase by *Penicillium echinulatum* on 7th day of incubation in SmF (Ritter et al. 2013). The low production of enzyme components of cellulase by *Penicillium* sp. on glucose supplemented medium was observed in the present study. Similarly, no cellulolytic activity was observed in culture filtrate of *Penicillium* sp. grown on Potato Dextrose Broth (Picart et al. 2007). Gautam et al. (2010) studied the production of cellulase (filter paper activity, β-endoglucanase and β-glucosidase) by *Aspergillus niger* on three different carbon sources such as glucose, cellulose and waste cellulosic material. Glucose containing media gave the highest mycelial weight of 1.294 mg/flask. Maximum cellulase enzyme activity (filter paper activity, endoglucanase and β-glucosidase) were obtained from the culture containing cellulose. Low levels of production of cellulase enzymes by organisms in media with glucose even in the present study could be attributed to repression of synthesis of the cellulase enzymes related to catabolism of alternate carbon such as cellulose (Ruijter and Visser 1997; Peterson and Nevalainen 2012; Gusakov and Sinitsyn 2012).

**Table 2:** Effect of supplementation of carbon source on cellulase production by *Penicillium* sp.

Carbon source	Dry weight of mycelial mat (mg/flask)	Protein content (mg/ml)	Cellulase		
			^a^FPase (FPU/ml)	^b^CMCase (U/ml)	^c^Β-glucosidase (U/ml)
Sarbose	420	2.17	3.53	8.23	1.16
Maltose	369	2.37	8.24	10.35	1.67
Sucrose	275	2.05	5.40	13.18	1.87
Lactose	497	2.78	9.12	24.70	2.32
Dextrose	412	2.45	5.18	10.35	1.04
Galactose	445	2.12	6.82	14.35	2.01
Cellobiose	338	1.92	-nd-	14.12	1.46
CMC	529	1.97	4.71	18.82	1.95
Control	378	2.28	5.99	11.99	1.98

Values represented in the table are averages of results of two experiments

*-nd-* not detected

^a^Filter paperase (FPase) is expressed in terms of filter paper units. One unit is the amount of enzyme in the culture filtrate releasing µmole of reducing sugar from filter paper per min

^b^Carboxymethyl cellulase (CMCase) is expressed in terms of units. One unit is the amount of enzyme releasing 1 µmol of reducing sugar from carboxymethyl cellulose per min

^c^One unit of β-glucosidase activity is defined as the amount of enzyme liberating 1 µmol of *p*-nitrophenol per min

Cellulase production on different nitrogen sources by *Penicillium* sp. is compared (Table 3). Among all these nitrogen sources (0.2 %) tested in this study, yeast extract is the best source followed by peptone for production of cellulolytic enzymes. Biomass, extracellular protein content yielded by *Penicillium* sp. were higher on organic nitrogen than on inorganic nitrogen and were correlated to cellulase production. Peptone enhanced CMCase activity in *Gliocladium virens,* and high β-glucosidase activity in *A. niger* and *A. terreus* (Gomes et al. 1989). Similarly, enhancement of cellulase production in *Volariella displasia* occurred in the presence of peptone (Guptha et al. 1996). The addition of 2 % w/w urea to soy hull in solid state fermentation by *Phanerochaete chrysosporium* enhanced the production of CM-cellulase and filter paperase (Jha et al. 1995). Incubation of urea in medium at high concentration of 0.525 g/l resulted in maximal production of cellulase by *Penicillium echinulatum* in SmF (dos Reis et al. 2015). High yields of protein content was attained by cultivation of *Chrysosporium* sp. and *Thielavia* sp. on Czapek medium containing beet pulp as carbon, and urea as nitrogen source (Bilai et al. 1985). Various researchers have shown that different organic and inorganic nitrogen sources such as yeast extract (Ganguly and Mukherjee 1995); soya meal (Gomes et al. 2000) and corn steep liquor (Hayward et al. 2000) influenced the cellulase production. Organic nitrogen substances had varied effects on production of individual enzyme components in cellulase system by *Polyporous* sp. (Nigam and Prabhu 1991). Peptone was found to be the most promising and effective nitrogen source for cellulase production by *Penicillium waksmanii* F10-2 (Han et al. 2009 **)**. Supplementation of NH_4_NO_3_ as the nitrogen source had the highest impact on cellulase production (Singhania et al. 2006).

**Table 3:** Effect of supplementation of nitrogen source on cellulase production by *Penicillium* sp.

Nitrogen source	Dry weight of mycelial mat (mg/flask)	Protein content (mg/ml)	Cellulase		
			^a^FPase (FPU/ml)	^b^CMCase (U/ml)	^c^Β-glucosidase (U/ml)
NH_4_Cl	150	1.08	-nd-	0.70	0.34
(NH_4_)_2_SO_4_	112	0.97	-nd-	-nd-	0.12
KNO_3_	165	1.00	-nd-	1.20	1.20
Peptone	475	2.07	1.35	8.64	3.68
Urea	405	1.38	0.15	3.36	1.74
Yeast extract	490	2.38	2.70	12.00	11.35
Control	378	2.28	5.99	11.99	1.98

Values represented in the table are averages of results of two experiments

*-nd-* not detected

^a^Filter paperase (FPase) is expressed in terms of filter paper units. One unit is the amount of enzyme in the culture filtrate releasing µmol of reducing sugar from filter paper per min

^b^Carboxymethyl cellulase (CMCase) is expressed in terms of units. One unit is the amount of enzyme releasing 1 µmol of reducing sugar from carboxymethyl cellulose per min

^c^One unit of β-glucosidase activity is defined as the amount of enzyme liberating 1 µmol of *p*-nitrophenol per min

High levels of CMCase (50–60 U/ml) and filter paperase (3 U/ml) along with maximum extracellular protein content were attained with the addition of corn steep liquor (Farid and El-Shaheed 1993). In addition, whey at low levels 0.2 % to the medium of cellulose and corn steep liquor stimulated cellulase production but higher concentrations inhibited cellulase production. The addition of skim milk powder at 0.2 % enhanced activities of exoglucanase and endoglucanase by *Trichoderma reesei* but had no influence on β-glucosidase activity (Patil et al. 1995). Organic forms of nitrogen such as yeast extract and peptone served as better nitrogen sources for production of cellulase in comparison to inorganic nitrogen forms in the present study.

The bioconversion of agro waste based lignocellulosic material to energy has gained much interest during the recent past. Low cost of enzyme production improves the economics, as the cost of enzymes constitutes a major part of the total cost. Lignocellulosics are abundant sources of carbohydrate, continually replenished by photosynthetic reduction of carbon dioxide by sunlight energy. Lignocelluloses are complex polymers consisting of cellulosic fibrous bundles encased in polymer of matrix of hemicellulose and lignin. Whether lignocelluloses could support cellulase production by *Penicillium* sp. was tested. Of all these lignocelluloses supplemented in the Dox medium, sawdust secretion maximal protein content along with highest titers of cellulolytic enzymes (Table 4). Growth of *Penicillium* sp. on medium with saw dust yielded biomass considerably high but lower than that obtained on wheat bran. However, wheat bran ranked the second in order in supporting cellulase production and secretion of extracellular protein content. Four species of *Cyathus* produced biomass of 4.3–4.5 g and a complete cellulase system on paddy husks (Alka-Gupta et al. 1999). Of the cellulosic materials tested, rice straw supplemented medium production of cellulase activity by *Penicillium* sp. (Picart et al. 2007). Hafiz Iqbal et al. (2010) investigated the potential of a filamentous fungus, *Trichoderma harzianum* for hyper-production of the most demanded industrial enzyme carboxymethyl cellulase using cheap and easily available agro-industrial residue wheat straw as growth supporting substrate under still culture solid state fermentation technique. According to the study of Alam et al. (2009) growth of *Trichoderma harzianum* T2008 on empty fruit bunches under SSF exhibited maximum FPase activity (8.2 IU/g) at 32 °C after 4 days of incubation in Erlenmeyer flask.

**Table 4:** Cellulase production on lignocelluloses by *Penicillium* sp.

Source	Dry weight of mycelial mat (mg/flask)	Protein content (mg/ml)	Cellulase		
			^a^FPase (FPU/ml)	^b^CMCase (U/ml)	^c^Β-glucosidase (U/ml)
Saw-dust	573	2.34	6.59	23.05	5.03
Rice-straw	341	1.36	4.47	13.88	3.47
Wheat bran	605	1.72	4.94	16.70	4.37
Paper	315	1.42	0.59	9.90	2.85
control	378	2.28	5.99	11.99	1.98

Values represented in the table are averages of results of two experiments

*-nd-* not detected

^a^Filter paperase (FPase) is expressed in terms of filter paper units. One unit is the amount of enzyme in the culture filtrate releasing µmol of reducing sugar from filter paper per min

^b^Carboxymethyl cellulase (CMCase) is expressed in terms of units. One unit is the amount of enzyme releasing 1 µmol of reducing sugar from carboxymethyl cellulose per min

^c^One unit of β-glucosidase activity is defined as the amount of enzyme liberating 1 µmol of *p*-nitrophenol per min

Mrudula and Murugammal (2011) reported maximum cellulase production by *Aspergillus niger* using coir waste as substrate. Utilization of 1 g of rice straw by successive cultivation of *Aspergillus ustus, Trichoderma* sp., *Botrytis* sp. and *Sporotrichum* sp. on rice straw and wheat bran in solid state fermentation gave production of 14 U of FPA, 22 U of CMCase and 48 U of β-glucosidase per gram of dry solids utilized in solid state fermentation (Duemas et al. 1995). Growth of *Chaetomium globosum* on oil palm empty fruit bunch fiber yielded titer of FPase (2.5 U/ml), CMCase (59 U/ml) and β-glucosidase (12 U/ml). Solid-state fermentation of coconut coir pith by *T. viridae* for 7 days produced Fpfase 4.7 U, CMCase of 12 U and P-glucosidase of 1.8 U per gram of dry solid (Muniswaran and Charyulu 1994). Growth of *A. niger, Pencillium citrinum, P. chysogenum* on modified Czapek Dox medium supplemented with wheat bran produced maximum extracellular Fpase, CMCase and β-glucosidase activity (EI-Shayeb et al. 1992).

Temperature highly influences the growth and enzymatic activities of organism. Many researchers have reported different temperatures for maximum cellulase production either in flask or in fermenter studies using *Aspergillus* sp. and T*richoderma* sp. suggesting that the optimal temperature for cellulase production also depends on the strain variation of the microorganism (Krishna 1999; Lu et al. 2003). The growth, extracellular protein content and cellulases production by *Penicillium* sp. grown at three different temperatures was monitored and are presented in Table 5. Among three tested temperatures, 30 °C is the better choice for cellulase activity along with growth and extracellular protein content. Fungal strain *P. sajor*-*caju* yielded highest activities of Endo-β-1, 4-glucanase, Exo-β-1, 4-glucanase and β-glucosidase to the tune of 18.98, 13.63 and 18.54 Units (μmol of glucose released/min/g substrate), respectively, at 25 °C (Pandit and Maheshwari 2012). Optimal temperature at 30 °C was observed for cultivation *of Aspergillus niger* on coir waste for production of cellulase (Mrudula and Murugammal 2011). Highest β-glucosidase activity by *T. viridae* in maize cobs medium incubated at 28 °C (Ye and Fields 1989) occurred. *T viridae* produced the highest level of cellulase on dried apple pomace under solid state fermentation incubated at 30 °C for 7-days (Bhalla and Joshi 1993). The optimum temperature for the production of CMCase by *Aspergillus* sp. was 37 °C (Asquieri and Park 1992; Kathiresan and Manivannan 2006; Devanathan et al. 2007). The increase in the culture temperature of thermophilic cultures of *Allesheria terretris* from 40 to 48 °C resulted in high cellulase production (Kvesitadze et al. 1986). High CM-cellulase and β-glucosidase activities were reported with *T. viridae, T harzianum* and *Gliocladium virens* in basal medium incubated at 30 °C (Gomes et al. 1989). Maximum CM-cellulase and filter paper activity were observed with *Ulocladium chartarum* in basal medium incubated at 30 °C among a range of temperature from 5 to 45 °C used in the study (Sallam et al. 1988). Similarly, optimum temperature was found to be 30 °C for cellulase production by *Penicillium* sp. in the present study. Ali et al. (1991) reported maximum yield of cellulase by *Aspergillus niger* Z10 strain and *A. terreus* at 40 °C, respectively in SSF.

**Table 5:** Effect of temperature on the production of cellulase by *Penicillium* sp.

Temperature E (°C)	Dry weight of mycelial mat (mg/flask)	Protein content (mg/ml)	Cellulase		
			^a^FPase (FPU/ml)	^b^CMCase (U/ml)	^c^Β-glucosidase (U/ml)
25 °C	293	1.02	-nd-	4.56	0.62
30 °C	457	2.12	2.03	9.83	1.04
37 °C	457	2.12	1.62	6.24	0.99

Values represented in the table are averages of results of two experiments

*-nd-* not detected

^a^Filter paperase (FPase) is expressed in terms of filter paper units. One unit is the amount of enzyme in the culture filtrate releasing µmol of reducing sugar from filter paper per min

^b^Carboxymethyl cellulase (CMCase) is expressed in terms of units. One unit is the amount of enzyme releasing 1 µmol of reducing sugar from carboxymethyl cellulose per min

^c^One unit of β-glucosidase activity is defined as the amount of enzyme liberating 1 µmol of *p*-nitrophenol per min

Among physical parameters, pH of the growth medium plays an important role by inducing morphological changes in microbes and in enzyme secretion. The pH change observed during the growth of microbes also affects product stability in the medium. The optimal pH varies with different microorganisms and enzymes. The effect of initial pH on cellulase production by *Penicillium* sp. in Czapek-Dox medium with 0.5 % (w/v) of cellulose powder was assessed. The results are represented in Table 6. *Penicillium* sp. produced maximum growth and secretion of extracellular protein when cultured at pH-5. This was reflected by yields of fungal mat (457 mg/flask) and (2.12 mg/ml) of extracellular protein in the culture filtrate at the end of 7th-days incubation. High activities of FPase (2.03 U/ml), CMCase (9.839 U/ml) and β-glucosidase (1.04 U/ml) were observed. Low activities of the above described enzymes were observed in the cultured flasks whose initial pH of the medium was set to pH-6.0 and pH-7.0, but extracellular protein content was little high in pH-7.0 than pH-6.0. Very low vegetative growth of 75 mg/flask and 0.2 mg/ml of extracellular protein content and no individual cellulolytic enzyme activities were recorded when the culture was grown on the medium with initial pH-3.0. No FPase, but 1.02 U/ml of endoglucanase and 0.18 U/ml of β-glucosidase was observed in the culture whose initial pH was set to pH-4.0. Only CMCase with measurable 1.153 U/ml was detected in the culture filtrate of the culture grown at pH-8.0. It was clear from the results that pH-5.0 was found to be maximal for the production of biomass and cellulase complex by *Penicillium* sp.

**Table 6:** Effect of pH on the production of cellulase by *Penicillium* sp.

Initial pH	Dry weight of mycelial mat (mg/flask)	Protein content (mg/ml)	Cellulase		
			^a^FPase (FPU/ml)	^b^CMCase (U/ml)	^c^Β-glucosidase (U/ml)
3	075	0.2	-nd-	-nd-	-nd-
4	217	1.95	-nd-	1.02	0.18
5	457	1.92	2.03	9.83	1.04
6	450	1.52	1.14	6.53	0.67
7	400	1.60	0.94	4.21	0.21
8	206	1.16	1.15	-nd-	-nd-

Values represented in the table are averages of results of two experiments

*-nd-* not detected

^a^Filter paperase (FPase) is expressed in terms of filter paper units. One unit is the amount of enzyme in the culture filtrate releasing 1 1.1 mol of reducing sugar from filter paper per min

^b^Carboxymethyl cellulase (CMCase) is expressed in terms of units. One unit is the amount of enzyme releasing 1 µmol of reducing sugar from carboxymethyl cellulose per min

^c^One unit of 13-glucosidase activity is defined as the amount of enzyme liberating 1 µmol of *p*-nitrophenol per min

The highest activities of Endo-β-1, 4-glucanase (17.65 U/g), Exo-β-1, 4-glucanase (13.49 U/g) and β-glucosidase (14.62 U/g) were obtained at pH of 5 (Pandit and Maheshwari 2012). The maximum cellulase activity was achieved when *Trichoderma viride* strains were cultivated in medium set to a range of pH 5–6; as pH increased up to 5.5, the hyper activities of exoglucanase (2.16 U/ml), endoglucanase (1.94 U/ml) and β-glucosidase (1.71 U/ml) were observed (Gautam et al. 2010). Similarly, Maheswari et al. (1993) made an observation that acidic pH-5.5 was found to be optimal for maximal cellulase production. The initial pH of the culture medium had marked effect on cellulase production by the different organisms. The production of cellulolytic enzymes by *Sacchobolus saccoboloides* (Magnelli et al. 1996), *Nectria cataliensis* (Pardo and Forchiassin 1998; Romero et al. 1999) was maximal at the initial pH of growth medium of 6.5. Higher level cellulolytic activity in respect of *Coriolus hirsitus* and *Coriolus versicolor* grown at pH-6.5 to 7.5 than at pH 3.0–4.0 was correlated to their higher growth (Dudehenko et al. 1988). The activity of the culture grown at pH-5.0 was fairly stable and optimum compared to other pH value with *Fusarium solani* (Bisen et al. 1982). The yield of cellulase by *Trichoderma harzianum* was improved at pH-5.0 (Rousses and Raimbault 1982). In the present study, pH was not controlled in the medium during the course of experiment. The yields of enzyme production might have been further improved with pH control during the course of the growth.

The effect of surfactants (sodium dodecyl sulphate, sodium deoxycholate, Triton X-100, Tween-20 and Tween-80) on the production of cellulase by *Penicillium* sp. was determined after 7 days of incubation in Table 7. Highest production of dry mass (514 mg/flask), and extracellular protein content (2.01 mg/ml) was recovered in the culture filtrate grown on Triton-X100 supplemented medium. The same culture filtrate exhibited maximum activities of FPase (3.088 U/ml), CMCase (11.999 U/ml) and 1.992 U/ml of glucosidase. No FPase activity was detected in other surfactants used in this study. Low amounts of dry mass in sodium dodecyl sulphate (350 mg/flask), extracellular protein content in sodium deoxycholate (1.23 mg/ml) were observed. Only 7.529 U/ml of CMCase and 0.903 U/ml of β-glucosidase activity were recorded in the presence of sodium deoxycholate. Among different non-ionic surfactants (Tween-20, Tween-80, and Triton X-100) and polyethylene glycol (PEG-600), Tween-80 yielded highest titers of exoglucanase, endoglucanase and cellobiase by *Nectria cataliensis* (Pardo 1996). This similar effect of Tween-80 on production of extracellular protein, in particular, cellulase by other organisms, *Trichoderma* (Reese and Maguire 1969; Domingues et al. 2000) and thermophile *Thermomonospora curvata* (Stutzenberger 1987) was observed. According to the study of Domingues et al. (2000), Tween-80 influenced the morphology of *Trichoderma reesei Rut* C-30 as well as the enzyme production. The stimulatory effect of surfactants may be a consequence of its action on cell membranes causing increase in permeability by promoting the release of cell-bound enzymes (Abdel-Fatah et al. 2012). On the other hand, Triton X-100 appeared to be the best surfactant for the production of cellulase in the present study.

**Table 7:** Effect of supplementation of surfactants source on cellulase production by *Penicillium* sp.

Source	Dry weight of mycelial mat (mg/flask)	Protein content (mg/ml)	Cellulase		
			^a^FPase (FPU/ml)	^b^CMCase (U/ml)	^c^Β-glucosidase (U/ml)
SDS	350	1.62	-nd-	9.88	1.66
SDO	410	1.23	-nd-	7.52	0.90
TritonX-100	514	2.01	3.08	11.99	1.99
Tween-80	492	1.74	-nd-	10.25	1.04
Tween-20	480	1.67	-nd-	9.88	2.84
Control			5.99	11.99	1.98

Values represented in the table are averages of results of two experiments

*SDO* sodium deoxycholate

*-nd-* not detected

^a^Filter paperase (FPase) is expressed in terms of filter paper units. One unit is the amount of enzyme in the culture filtrate releasing 1 1.1 mol of reducing sugar from filter paper per min

^b^Carboxymethyl cellulase (CMCase) is expressed in terms of units. One unit is the amount of enzyme releasing 1 µmol of reducing sugar from carboxymethyl cellulose per min

^c^One unit of 13-glucosidase activity is defined as the amount of enzyme liberating 1 µmol of *p*-nitrophenol per min

Growth of *Penicillium* sp. on optimized medium (cellulose 0.5 %, lactose 0.5 %, sawdust 0.5 %, yeast extract 0.2 %, and Triton-X 100 0.015 % at pH 5.0, temperature 30 °C) in submerged fermentation in the present study yielded the production of Fpase, CMCase and β-glucosidase to the extent of 8.7, 25 and 9.2 U/ml, respectively, which was 9.2, 5.9 and 43.8-folds higher than titers of the respective enzymes obtained on unoptimized medium with the same culture. The extracellular protein content (4.5 mg/ml) and biomass (520 mg/flask) was also increased under optimal conditions over unoptimized conditions. Similarly, optimization of medium components resulted in enhancement in production of cellulose enzymes by *Penicillium echinulatum* by 20–80 % over unoptimised medium (dos Reis et al. 2015) It is clear from the results of the present study that *Penicillium* sp. secreted cellulase with high β-glucosidase activity in SmF and the ratio of β-glucosidase to Fpase of *Penicillium* sp. is greater than one and will be useful in saccharification process of lignocellulosic biomass for biofuel production.
